# Spiritual Well-Being and Quality of Life of Iranian Adults with Type 2 Diabetes

**DOI:** 10.1155/2014/619028

**Published:** 2014-01-29

**Authors:** Najmeh Jafari, Ziba Farajzadegan, Amir Loghmani, Mansoureh Majlesi, Noushin Jafari

**Affiliations:** ^1^George Washington Institute for Spirituality and Health, School of Medicine and Health Sciences, George Washington University, Washington, DC 20036, USA; ^2^Community Medicine Department, School of Medicine, Isfahan University of Medical Sciences, Isfahan 81745-313, Iran; ^3^Vice-Chancellery for Treatment, Isfahan University of Medical Sciences (IUMS), Isfahan 81656-47194, Iran; ^4^Anesthesiology Department, School of Medicine, Isfahan University of Medical Sciences, Isfahan 81745-313, Iran

## Abstract

*Introduction*. Diabetes is a major public health problem. Little is known about the spiritual well-being and its relationship with quality of life (QOL) in Iranian Muslim patients with diabetes. This study investigated the spiritual well-being and QOL of Iranian adults with type 2 diabetes and the association between spiritual well-being, QOL, and depression. *Methods*. A cross-sectional study was done among 203 patients with type 2 diabetes mellitus in Isfahan, Iran. Quality of life and spiritual well-being were measured using the functional assessment of chronic illness therapy-spiritual well-being (FACIT-Sp). Depression was assessed using the Patient Health Questionnaire-2 (PHQ-2). Descriptive analysis, Pearson's correlation, and multiple regression analysis were performed for statistical assessment. *Results*. The mean QOL was 61.00 (SD = 9.97) and the mean spiritual well-being was 30.59 (SD = 6.14). Sixty-four percent of our studied population had depressive disorders. There was a significant positive correlation between all QOL subscales and meaning, peace, and total spiritual well-being score. *Conclusion*. The results of this study showed poor QOL and spiritual well-being and high prevalence of depression in Iranian patients with type 2 diabetes compared to other studies' findings especially western studies. This indicates the need for psychosocial and spiritual support in caring for Iranian patients with diabetes.

## 1. Introduction

Diabetes is a serious public health problem with an increasing incidence in Middle East countries as well as Iran [1]. Five of the 10 world's highest national prevalences of diabetes occur in Middle East countries, that might be expected to increase in the coming decades [1, 2]. The last nationally representative report of the burden of diabetes in Iran showed a high prevalence of diabetes (8.7%) in Iranian population, which is estimated to rise to 9.8% in upcoming decades [3].

Diabetes is one of the most psychologically demanding chronic medical disorders and is often associated with several psychiatric disorders [4]. Diabetic patients are about twice as likely as people without the condition to have anxiety, depression, and serious psychological problems [5–7]. Emotional distress may influence outcomes in terms of glycemic control, adherence to medical treatment, cost of care, and mortality [8].

The interface between poor physical health and poor mental health affects quality of life (QOL) of diabetic patients [9]. QOL is a broad, multifaceted concept [10]. It incorporates the individual's subjective perception of physical, emotional, cognitive, social, and spiritual domains of an individual's life [11]. Several studies indicated that diabetic patients have reduced QOL compared to general population in the same age group [12, 13], and their QOL decreases with diseased progression and complications [14, 15].

Among several components of QOL, spirituality receives more attention in recent years [16]. Spirituality is defined as “the aspect of humanity that refers to the way individuals seek and express meaning and purpose and the way they experience their connectedness to the moment, to self, to others, to nature, and to the significant or sacred” [17]. There is a strong association between spirituality and coping with chronic medical disease [18], willingness to live [19], reducing anxiety and depression [20], and improving quality of life [21].

The importance of addressing spirituality in diabetes management is indicated in several studies. Previous studies on African American population showed that there is a positive relationship between spiritual well-being and coping with diabetes, glycemic control, and self-management. [22–24]. However, there is minimal information regarding the spirituality of patients of different cultures such as Iranian Muslim patients.

In two qualitative studies from Iran, the role of spiritual beliefs in coping with diabetes and patients' empowerment is indicated [25, 26]. However, there is still lack of evidence regarding measuring the spiritual well-being and assessing its association with QOL using a standard tool. Different cultural groups and religious affiliations may emphasize different aspects of their QOL and spiritual well-being [27]. Assessing the local perspectives by international instruments will provide an opportunity for cross-cultural comparisons and developing the best interventions based on the needs of patient with diabetes.

The aims of this study were to describe the spiritual well-being and QOL of Iranian adults with type 2 diabetes and to investigate the association between spiritual well-being, QOL, and depression among Iranian adults with type 2 diabetes.

## 2. Materials and Methods

This was a cross-sectional study, which was conducted in two diabetes care institutes in Isfahan, Iran, from January 2013 to April 2013. These institutes are main diabetes care centers located in two different geographic areas of Isfahan city, covering more than 10,000 diabetes patients with diverse socioeconomic characteristics. The target population was patients with type 2 diabetes who had registered in these institutes and consented to participate. People aged 18 or above with a definitive diagnosis of type 2 diabetes, as confirmed by a physician, with or without complication and the ability to read and write Farsi, were enrolled in the study. The exclusion criteria was any documented diagnosis of end-stage renal disease, psychotic disorder, dementia, or blindness.

### 2.1. Instruments

To assess the spiritual well-being and QOL of participants, we used the functional assessment of chronic illness therapy-spiritual well-Being (FACIT-Sp) scale. This is a valid and reliable instrument that was developed in the 1990s to provide an inclusive measure of spirituality in research and clinical practice [28, 29]. This questionnaire assesses spirituality well-being as well as QOL regardless of religious or spiritual tradition [29]. It consists of a core general questionnaire for measuring QOL and an additional scale for measuring spirituality. The core general questionnaire that measures QOL (FACT-G) is composed of four subscales: physical well-being (PWB = 7 items), social/family well-being (SWB = 7-items), emotional wellbeing (EWB = 6 items), and functional well-being (FWB = 7 items) [30]. The additional scale for measuring spirituality contains 12 items and three subdomains (peace, meaning, and faith) [29]. The FACIT-Sp is self-administered and uses a 5-point Likert-type scale (0 = not at all; 4 = very much) and the score range of 0–48 [29]. The higher score represents the better spiritual well-being. All three scales have high internal consistency (Cronbach's alpha for total scale 0.87, for meaning/peace subscale 0.81, for faith subscale 0.88). This questionnaire is translated and validated to Persian by authors and we found that the Persian version of the FACIT-Sp scale is a reliable and valid tool for the clinical assessment of, and research into, the spiritual well-being of Muslim Iranians [31].

We evaluated depression using the 2-item patient health questionnaire depression module, the PHQ-2 [32]. This tool inquires about the frequency of depressed mood and anhedonia over the past 2 weeks, scoring each as 0 (not at all) to 3 (nearly every day); thus, the PHQ-2 score can range from 0 to 6. PHQ-2 cutoff score of ≥3 had the best tradeoff between sensitivity (79%) and specificity (86%) for any depressive disorder. This tool appears promising as a brief sensitive and specific tool for detecting and monitoring of depression [33].

To assess the glycemic control of participants we consider glycosylated hemoglobin HbA_1c_ as a reliable index of long-term glycemic control in patients with diabetes. An elevated HbA_1c_ indicates poor long-term glycemic control [34]. In this study HbA_1c_ level ≤ 7 was considered as controlled diabetes and HbA_1c_ level > 7 as uncontrolled diabetes.

Patients were randomly sampled using IBM SPSS Statistics for Microsoft Windows, Version 21. Demographic information (age, marital status, education, and occupation) was collected through a self-administered questionnaire. Clinical data including laboratory and concurrent chronic disease were extracted from medical records. After oral and written consent, participants were instructed to read the brief directions at the top of the questionnaire. After confirming the participants' correct understanding, they were encouraged to complete every item in private. A total of 203 participants completed the FACIT-Sp, PHQ-2, and demographic information questionnaire.

### 2.2. Statistical Analysis

The sample was described using means and standard deviations for quantitative variables and relative frequencies and percentages for categorical variables. *t*-test analysis was used to compare the mean of FACIT-Sp and PHQ-2 scores in two groups (controlled diabetes and uncontrolled group).

The bivariate relationship between spiritual well-being, QOL subscales, depression, and diabetes control (HbA_1c_) was assessed by calculating Pearson correlation coefficients. Variables were selected for inclusion in the multiple regression model based on theoretical importance as well as significance in bivariate analyses. Multiple regression analysis was used to assess the predictor role of the 12-item spiritual well-being subscale of the FACIT-Sp subdomains (peace, meaning, and Faith) on determinants of QOL. The FACT-G total scores and its subdomains (PWB, SWB, EWB, and FWB) considered as the dependent variables [35] and the independent (predictor) variables (peace, meaning, and faith, PHQ-2 and HbA_1c_) were entered in blocks.

Collinearity diagnostics were performed by means of the variance inflation factor (VIF) for each independent variable entered in the regression equations. A VIF > 10 was considered as positive multicollinearity [36]. The level of significance was set at *P* > 0.05, and all tests were two-tailed. Data were analyzed using SPSS (version 21) for Windows.

## 3. Results

Over a six-week-period, 223 patients met the inclusion criteria and were recruited. Twenty patients were excluded because of dementia (*n* = 6), psychotic disorders (*n* = 3), and declining to take part (*n* = 11). The mean age of all participants was 55.42 (SD = 10.67) with a range of 18–87 years. The majority of participants were female (69.5%) and married (95.1%). Seventy-two percent of participants were housewives and 8.4% were retired. Seventy-six percent of them were educated below high school diploma, 29 patients received their diploma, and 18 patients had master degree from the university. No gender differences were detected regarding education. All patients identified themselves as Muslim. There was no statistically significant difference regarding demographic characteristics between patients who participated and who declined (*P* > 0.05).

The mean of fasting blood sugar (FBS) was 163.26 (SD = 62.14). Among our participants, 76 diabetic patients (37.4%) were in the controlled group (HbA_1c_ ≤ 7) and 127 (63%) were in uncontrolled group (HbA_1c_ > 7).

The mean score of FACT-G was 61.00 (SD = 9.97). The mean spiritual well-being (FACIT-Sp12) score was 30.59 out of 48 (SD = 6.14) with the highest mean score in the faith subscale (mean = 10.78, SD = 2.89) in comparison to the other subscales. The mean PHQ-2 score was 4.7 (S.D. = 1.5). Considering the cutoff score of ≥3, 63.5% of our studied population had depressive disorders.

In one-way ANOVA, there was no statistically significant difference in QOL, spiritual well-being, and depression scales regarding demographic status (*P* > 0.05). Among FACIT-Sp subdomains, the mean of physical well-being, emotional well-being, functional well-being, and peace scores was higher in controlled diabetes group in comparison to uncontrolled group (*P* < 0.05). People in uncontrolled group had higher score in PHQ-2 score of depression (*P* = 0.047).  Table 1 shows participants' quality of life as well as spiritual well-being and depression scores in two groups.

Table 2 lists the bivariate Pearson correlation coefficients between the QOL, spirituality, and depression measures. There was a statistically significant positive correlation between all QOL subscales (physical, social, emotional, and functional well-being) and meaning, peace, and total FACIT-Sp-12 scores. HbA_1c_ was negatively associated with physical well-being, emotional well-being, functional well-being, and peace, otherwise, positively correlated with depression. Depression was also significantly correlated with all QOL and spiritual well-being subscales and this association was highest with emotional well-being (*r* = −0.56, *P* < 0.001), physical well-being (*r* = −0.52, *P* < 0.001), and peace (*r* = −0.48, *P* < 0.001).

In regression analyses, after evaluating the correlations among the independent variables, no multicollinearity problem was detected. Meaning and peace subscales of FACIT-Sp12 were significantly associated (*P* < 0.05) with total QOL. Meaning, peace, faith, and depression were significantly associated with physical well-being (*R* ^2^ = 0.37, *P* < 0.005). In each of these analyses, the meaning and peace subscales were significantly associated with the subdomains of QOL. The faith subscale, on the other hand, was only associated with physical well-being and did not provide an independent contribution to prediction of other domains. Furthermore, HbA_1c_ did not contribute significantly to any of these outcomes (Table 3).

## 4. Discussion

This study was designed to assess spiritual well-being and QOL together with depression in a population of Iranian people with type 2 diabetes and to explore their possible associations. Our studied population had poor spiritual well-being and quality of life in comparison to the normative data of the general U.S. population [37] but similar to another study from Iran in patients with cancer [31].

In our Muslim population, the highest score of spiritual well-being was related to faith subdomain of FACIT-Sp12. This coheres with other studies in Muslim population [38, 39]. The “faith” component of FACIT-Sp-12 is most often associated with religion and religious beliefs [40], but spirituality extends beyond religion and is independent of commitment to a particular religion or doctrine [41]. Spirituality is experiencing transcendent meaning and purpose in life as well as sense of connectedness. Several studies indicated the importance of spirituality in coping with disease, better QOL, and hopefulness [42–45]. In our study meaning and peace subscales of FACIT-Sp12 were significantly associated with all aspects of QOL. In regression analyses, higher meaning and peace were related to better physical, social, emotional, and functional well-being as well as total QOL, whereas higher faith was only associated with physical well-being. These results are consistent with our study on Iranian patients with breast cancer, which showed that meaning and peace are more robust indicators of QOL than faith. This may be due to negative religious coping (e.g., belief that one's illness is God's punishment or abandonment) in patients with chronic illnesses [46]. However, this result is not specific to Iranian population and is replicated in several international studies. A large Australian study on 449 cancer patients found that the meaning/peace component being more highly related to QOL than the faith component [47]. Similarly, in two longitudinal studies, Yanez et al. showed that meaning and peace act as a positive resource for cancer survivors, but faith may serve to facilitate or even hinder positive adjustment [48]. In another study Canada et al. examined the 3-factor model for FACIT-SP-12 in two hundred and forty females previously diagnosed with cancer. They found that the peace factor was only correlated with mental health scores, meaning it was associated with both physical and mental health scores, and faith was negatively associated with mental health scores [49]. Edmondson et al. examined the role of religion in providing a sense of meaning and concluded that “religious is beneficial to the degree to which it facilitates the creation and maintenance of meaning, coherence, and purpose. Conversely, if religious beliefs and practices fail to provide meaning or provide meaning that is destructive (i.e., God no longer cares for me), they are ineffectual or detrimental to well-being” [50]. However, many Islamic scholars believe that there is no distinction between religion and spiritual concepts, and spirituality is meaningless without religious thoughts and performances [51]. Coherent with this, Islamic clergy men are included in some hospitals in Iran to provide the religious care to patients. The main aspect of this model of care is to help patients to do the Islamic rituals appropriately, which is far away by the spiritual care model that includes an interdisciplinary management to address all dimensions of care, including the spiritual, religious, and existential as well as physical and psychological.

Diabetes destroys not only the physical well-being of the patients but also threatens the social, functional, and emotional well-being of the patients. This condition causes the patients to ask about themselves, their purpose, and their meaning in life. Victor Frankl in his famous book “Man's search for meaning” states “Human is not destroyed by suffering; he is destroyed by suffering without meaning” [52]. Meaning has been assessed in terms of the sense of purpose in life, productivity, and reason for living [29]. This sense of meaning helps the patients to cope with their disease, reframing their lives, having an optimistic look on life and a “fighting spirit” against their disease [53, 54]. Previous studies on diabetic patients indicated that higher spiritual well-being is associated with lower HbA_1c_ and better adjustment to disease [22]. The findings of this study reflects that the sense of inner peace and intrinsic strength may guard against negative feelings and probably result in maintaining higher self-care behaviors and thus, greater glycemic control in those with diabetes. This highlights the need for considering spiritual issues in caring of the diabetic patients [55].

In our study controlled diabetes group (HbA_1c_ level ≤7) has better QOL and spiritual well-being in comparison to uncontrolled group (HbA_1c_ level >7). This is in line with the results of other studies indicating significant association of spiritual well-being and diabetes control [22, 23, 43]. A qualitative study on 70 African American women with type 2 diabetes showed the influence of spirituality in self-management of people with type 2 diabetes resulted in diabetes control [56].

In our studied population, individuals in the uncontrolled group had worse QOL. Diabetes can exert a negative impact on QOL [12]. Since diabetes is a chronic lifelong disease, patients with diabetes must deal with their disease all day. Medical therapy, diabetes complications, episodes of hypoglycemia, and fear of long term consequences may lead to reduced quality of life [57]. Furthermore, psychosocial toll of living with diabetes and psychiatric disorders such as depression were shown to be associated with poorer QOL [58].

Depression and diabetes are significantly connected to each other [59]. In this study, 63.5% of our studied population had depressive disorders. The prevalence of depression in our diabetic sample was higher than the range of 2%–9.5% in Iranian adults [60–62] as well as the rate of 8.3–28.8% of the diabetic patients in the United States [63]. However, the estimates of depression prevalence among individuals with diabetes appear to be higher in developing countries [64]. In a single point cross-sectional study done in India, depression was diagnosed in 43.34% [65]. In a study done in Pakistan, depression was found in 43.5% of patients with diabetes [66]. In another study from Iran, Khamseh et al. found major depression in 71.8% of a sample of 206 patients with type 1 and type 2 diabetes [67]. Other reports from Iran using different tools for depression showed high rates of depression in people with diabetes [68–70]. The higher co-incidence of depression in these populations may be due to the lower socioeconomic status that is a known risk factor for depression [71]. Furthermore, the high prevalence of depression in our sample may be due to use of a screening tool which may detect psychological distress rather than clinical depression and so may increase rates of disorder [72]. The results of our study showed that depression is negatively associated with all aspects of quality of life and spiritual well-being. In the regression analysis, depression was a significant predictor of physical and emotional well-being. This coheres with the results of previous studies that showed a moderate, negative association of depressive symptoms on physical and mental health [73–75], indicating the importance of detecting and managing depressive symptoms in diabetes care.

Our study, while having much strength, involved some limitations that should be mentioned. Our findings cannot be generalized to the general population of the patients with diabetes because sicker and older people with type 2 diabetes did not respond to the survey. Participants were from two clinics populations, and there was no general population comparison group to compare. Also, it is important to be aware that FACIT-Sp is not a diabetes specific tool to assess the quality of life and spiritual well-being. In this study we treat the spiritual well-being as the predictor variable. However the developers of this instrument believe that “… under certain conditions, spiritual well-being may function as a process variable or even as a dependent or outcome variable.” Furthermore we did not assess the religious coping pattern in our participants. More research, particularly longitudinal, is warranted to direct the causation between spiritual well-being, QOL, and depression in diabetic patients and to examine the feasibility of deriving a spiritual history in diabetes care.

Despite these limitations, the current study appears to be unique in that we assessed the QOL, and spiritual well-being and their association among diabetic patients in an Islamic context.

## 5. Conclusion

The results of this study showed poor QOL and spiritual well-being and high prevalence of depression in Iranian patients with type 2 diabetes compared to other studies' findings, especially Western studies. This indicates the need for psychosocial and spiritual support in caring for Iranian patients with diabetes.

## Figures and Tables

**Table 1:** FACT-G, FACIT-Sp12, and PHQ-2 scores of two groups (controlled and uncontrolled diabetic patients).

Domains	Group	Mean	Std. deviation	*P* value
Physical well-being	Controlled	17.61	6.18	0.033
	Uncontrolled	15.73	5.99	
Social/family well-being	Controlled	16.39	5.18	0.482
	Uncontrolled	15.84	5.52	
Emotional well-being	Controlled	10.80	5.38	0.045
	Uncontrolled	9.27	5.09	
Functional well-being	Controlled	19.55	4.24	0.004
	Uncontrolled	17.52	5.01	
Meaning	Controlled	10.97	3.11	0.214
	Uncontrolled	10.44	2.76	
Peace	Controlled	10.30	2.49	<0.001
	Uncontrolled	8.46	2.68	
Faith	Controlled	11.03	2.68	0.339
	Uncontrolled	10.63	3.00	
FACIT Sp12-score	Controlled	32.31	6.34	0.002
	Uncontrolled	29.55	5.79	
FACIT Sp-score	Controlled	95.15	13.88	0.005
	Uncontrolled	89.45	13.64	
PHQ-2 score	Controlled	2.73	1.87	0.047
	Uncontrolled	4.22	1.70	

HbA_1c_ level ≤ 7: controlled diabetes (*n* = 76).

HbA_1c_ level > 7: uncontrolled diabetes (*n* = 127).

**Table 2:** Pearson's correlation coefficients between FACT-G, FACIT-Sp12, and PHQ-2.

	Physical well-being	Social well-being	Emotional well-being	Functional well-being	Peace	Meaning	Faith	Spiritual well-being	PHQ-2	HbA_1c_
Physical well-being	1	0.245**	0.604**	0.381**	0.433**	0.430**	0.476**	0.376**	−0.522**	−0.159**
Social Well-being		1	0.351**	0.448**	0.475**	0.400**	0.009	0.409**	−0.315**	−0.054
Emotional well-being			1	0.418**	0.492**	0.558**	0.107	0.534**	−0.565**	−0.141*
Functional well-being				1	0.564**	0.601**	0.189**	0.626**	−0.408**	−0.153*
Meaning					1	0.498**	0.089	0.739**	−0.460**	−0.002
Peace						1	0.237**	0.797**	−0.476**	−0.253**
Faith							1	0.619**	−0.203**	−0.127
Spiritual well-being								1	−0.511**	−0.213**
PHQ-2									1	0.230**
HbA_1c_										1

**Correlation is significant at the 0.05 level (2-tailed).

*Correlation is significant at the 0.05 level (2-tailed).

**Table 3:** Association of FACT-G and its subdomains with spiritual well-being, depression, and HbA_1c_.

Predictors	Dependent variables									
	Physical well-being		Social well-being		Emotional well-being		Functional well-being		FACT-G	
	*β*	*P*	*β*	*P*	*β*	*P*	*β*	*P*	*β*	*P*
Meaning	0.182	**0.010**	0.339	**0.000**	0.179	**0.006**	0.347	**0.000**	0.369	**0.000**
Peace	0.195	**0.007**	0.217	**0.005**	0.321	**0.000**	0.383	**0.000**	0.253	**0.001**
Faith	0.192	**0.001**	0.087	0.171	0.053	0.335	0.053	0.329	0.111	0.061
PHQ-2	−0.374	**0.000**	−0.075	0.311	−0.344	**0.000**	−0.046	0.466	−0.111	0.107
HbA_1c_	−00.049	00.419	−0.006	0.923	−0.012	0.824	−0.040	0.475	−0.052	0.387

*R* ^ 2^	**0.374**		**0.270**		**0.454**		**0.461**		**0.368**	
*F *	**23.56**		**14.61**		**32.76**		**33.70**		**22.92**	

*β*: standardized beta; *P*: *P* value; method: enter.

The bold *P* values show the significant relationships among aspects of spirituality and QOL. Meaning and peace were significantly associated with all aspects of QOL.
